# Haze pollution reduction in Chinese cities: Has digital financial development played a role?

**DOI:** 10.3389/fpubh.2022.942243

**Published:** 2022-08-24

**Authors:** Chunkai Zhao, Bihe Yan

**Affiliations:** ^1^College of Economics and Management, South China Agricultural University, Guangzhou, China; ^2^School of Urban and Regional Science, Institute of Finance and Economics Research, Shanghai University of Finance and Economics, Shanghai, China

**Keywords:** digital financial development, haze pollution, PM_2.5_, exogenous shock, Chinese cities

## Abstract

Based on the exogenous shock of digital financial development in China in 2013, a difference-in-differences (DID) model is set up in this paper to investigate the causal relationship between digital financial development and haze pollution reduction. The finding of the paper is that a one standard deviation increase in digital finance after 2013 decreases the PM_2.5_ concentrations by 0.2708 standard deviations. After a number of robustness checks, like placebo tests, instrumental variable (IV) estimations, eliminating disruptive policies, and using alternative specifications, this causal effect is not challenged. In addition, this paper explores three potential mechanisms of digital finance to reduce haze pollution: technological innovation, industrial upgrading, and green development. Moreover, the heterogeneous effects signify that the usage depth of digital finance works best in haze pollution reduction. Digital finance has more positive effects in cities in the north and those with superior Internet infrastructure and higher levels of traditional financial development. However, the quantile regression estimates suggest that for cities with light or very serious haze pollution, the positive impact of digital finance is limited. These findings supplement the research field on the environmental benefits of digital finance, which provides insights for better public policies about digital financial development to achieve haze pollution reduction.

## Introduction

China's rapid economic growth, industrialization, and urbanization have exacerbated the severe air pollution problems, with increasing negative impacts on human health and social development (1–3). Since 2013, the World Health Organization has been tracking air quality to assess its impact on heart disease, lung cancer, and other respiratory diseases. In 2015, air pollution was responsible for more than 1.1 million deaths in China^1^. Apart from its detrimental effects on human health and the environment, air pollution can also result in significant economic loss. In China, it is estimated that the loss of labor supply due to air pollution caused a 1.34% drop in real GDP in 2007, with a GDP loss of approximately 361.468 billion Yuan (4).

China, as the world's largest developing country and haze pollutant emitter (5), has taken many measures to reduce air pollution (6), including strengthening environmental regulations (5) and enforcing traffic restrictions (7). One of the most prominent measures is to strengthen the use of financial instruments. These financial tools include the implementation of green credit policies and the opening of the national carbon emissions trading market. In the Industry 4.0 era, financial support is the basis for achieving sustainable development (8). As a result of the development of digital technology, digital finance has become not only a critical link in the financial system, but also an important growth point for the green economy in industry 4.0 (9).

Digital finance is usually used to refer to the financial industry's digitization, which encompasses all electronic products and services in the financial sector (10–12). Digital finance is defined as “financial services made available *via* cell phones, computers, the Internet, and cards connected to a secure digital payment system” (13). In general, any digital financial service consists of three critical components: the digital trading platform (the Internet), the retail agent, and the device used by the consumer and the agent to access the service online (most frequently a cell phone). In June 2013, Alipay launched Yu'E Bao, and then in November, the Third Plenary Session of the 18th Central Committee of the Communist Party of China (CPC) officially proposed “developing digital finance.” Therefore, it is commonly considered that 2013 was the first year of digital finance development in China (14). Resulting from the rapid development of third-party online payment platforms, represented by Alipay (15–17), China's digital finance has grown rapidly (18, 19). According to the report released by the Institute of Digital Finance, Peking University, and Ant Financial Services Group^2^, the average digital finance index (DFI)^3^ in Chinese prefecture-level cities has risen from 51.92 in 2011 to 230.73 in 2018, representing a 23.75% annual growth rate. Globally, the rapid rise of digital finance has attracted the attention of scholars and institutions [e.g., Shen et al. (12); Chen and Zhao (15); Wan et al. (19); Zhong (20)].

Current studies have concentrated on the macroeconomic implications of digital finance, including economic growth (12, 13); technological innovation (21); and traditional financial market shocks (22). There have been studies exploring the impact of digital finance on microeconomic aspects such as consumption structure (23), industrial structure (11, 16), and household welfare (15, 24). Additionally, some studies have been interested in the environmental impact of digital finance in recent years. One strand of literature looked at how digital finance affects carbon emissions, and these findings backed up the idea that digital finance works to cut carbon emissions (20, 25–27). Another category of studies highlighted the beneficial contribution of digital finance to sustainable development (20, 24, 28).

In contrast to the above studies, we pay close attention to the causal relationship and potential mechanisms between digital financial development and haze pollution reduction in China. However, there are some studies that are similar to our work. Specifically, Wan et al. (19) and Wen et al. (29) concerned about the influence of digital finance on industrial pollutant emissions, such as industrial sulfur dioxide. Although they argued that digital finance can aid in reducing industrial pollutant emissions in Chinese cities, there was a threshold effect. Moreover, Wang et al. (30) and Yang et al. (31) found a negative impact of digital finance on PM_2.5_ concentrations in China, but they did not analyze and examine the underlying mechanisms in detail. More importantly, the identification of the causal effect of digital financial development on haze pollution is not properly addressed in these previous studies.

To address these concerns, we employ the difference-in-differences (DID) approach to identify the causal relationship by using an exogenous shock of digital financial development in China in 2013 (32, 33), which effectively addresses the endogeneity issue. In 2013, mobile payments, represented by Alipay, are the dominant digital financial tool in China. With the popularity of e-commerce and the launch of Yu'E Bao, digital finance rapidly spread in China, and Alipay subsequently became the most used third-party online payment tool in the world^4^. Given the representativeness and importance of Alipay, we use DFI to represent digital finance, with the raw data of DFI coming from Alipay.

Our DID estimates support the idea that digital financial development can help curb haze pollution in China. The parallel trend tests illustrate that using this exogenous shock in 2013 is quite convincing. In addition, our results remain robust after excluding other disruptive policies, like Smart Cities, the Air Pollution Prevention and Control Action Plan (APPCAP), and Broadband China. We also confirm this causal effect by using the instrumental variable (IV) method, placebo tests, alternative specifications, and county-level data. Furthermore, we test the mechanisms by which digital financial development curbs haze pollution in China, including technological innovation, industrial upgrading, and green development.

The following contributions are made by this paper. First, we explore the positive impact of digital finance on haze pollution reduction and seek to identify the causal effects through an exogenous shock for digital finance, which further complements the research field on environmental benefits of digital finance. Second, this paper explores the mechanisms by which digital financial development reduces haze pollution in several ways, which not only provides insights for subsequent studies, but also informs the development of relevant public policies to exploit the pollution control effect of digital finance. Third, our heterogeneity analysis implies that the positive impact of digital finance on reducing haze pollution in different types of cities is inconsistent. Because of these, it is especially important to use digital finance to help control air pollution in accordance with local conditions.

The remaining sections of this paper are as below. Section 2 reviews the literature and develops hypotheses. Section 3 introduces the institutional background. Section 4 explains the methodology and variables. Section 5 presents and analyzes the empirical results. Section 6 concludes and discusses.

## Literature review and hypothesis development

Numerous academics have already examined finance's impact on the environment and pollution (34–36). However, as fintech and digital finance have developed at a breakneck pace in the past few years, the environmental benefits of digital finance have become a hot topic among academics. The impact of fintech on the environment, pollutant emissions, green growth, and energy efficiency is more abundantly studied than digital finance (18, 37, 38). These findings suggest that fintech, both globally (39) and in China (40), can not only reduce pollution, but also promote sustainable economic and ecological development. Also, studies on OECD countries (41) and Switzerland (42) have drawn the same conclusion.

Compared to fintech, digital finance is more focused on the widespread digitalization of the financial sector (13, 15). Additionally, some studies have demonstrated that the Internet could reduce air pollution and promote environmental sustainability (20, 43). Thus, digital finance, based on fintech and the Internet, is also likely to have positive environmental benefits. Nowadays, several studies have recognized the positive role of digital finance in reducing carbon emissions (19, 29). By curbing pollutants such as SO_2_ and PM_2.5_, digital finance can potentially improve air quality and promote sustainable development (30, 31). As mentioned above, we formulate our first hypothesis:

H1: Digital finance has a dampening effect on air pollution.

According to existing literature, digital financial development may curb haze pollution in the following three ways: technological innovation, industrial upgrading, and green development.

First, financial markets provide the necessary funds for innovative activities, which can have a direct impact on technological innovation and, consequently, on the development of new-energy and green technology (31). Due to restrictive competition and credit discrimination, financial resources cannot be optimally allocated in traditional financial markets, creating financial constraints on corporations (44), and impeding their capacity for green innovation (45). In comparison with traditional finance, digital finance alleviates information asymmetry and makes access to financial services more convenient, which has made it easier for small and medium-sized green businesses to get financial services (11, 46). Digital finance has effectively reduced the credit risk of banks, broadened the financing channels of enterprises (47), and eased their financial constraints, hence enhancing their technological innovation, promoting green technology innovation, and improving energy and environmental performance (10, 11, 48, 49). Furthermore, digital finance broadens the pool of investors and services, increasing funding volume and distributing the risk associated with innovation across a wider group. As a result, digital finance is more willing and able to enter high-risk innovation areas, enhancing green innovation (10, 17). Moreover, green innovation has a positive effect on the environment and cut down on haze pollution (5, 45, 50). Thus, the second hypothesis is put forth:

H2: Digital finance is likely to control air pollution through technological innovation effect.

Second, digital finance has sped up the upgrading of industrial structure in different ways, including expanding the range and depth of financial services and optimizing resource allocation (11, 51). More specifically, digital finance can assist in upgrading the industrial structure by improving the credit system and fostering the flow of funds to high-quality industrial projects (11, 52). In another words, digital finance helps to promote the industrial structure. Digital finance, according to Qu et al. (51), not only boosted the development of China's tertiary sector, but also expedited the greenization of industrial structure. Additionally, some studies found that digital finance benefits the transformation and upgrading of traditional manufacturing and agriculture. Chang et al. (53) conducted a review of the literature on digital finance and discovered that digital finance can promote the transition from traditional manufacturing to green manufacturing, affirming the positive effect of digital finance on industrial green upgrading. Using data from listed enterprises in China, Chen and Zhang (16) demonstrated empirically that digital finance greatly facilitated manufacturing servitization and promoted green manufacturing development. Simultaneously, because of the prevalence of digital trading platforms and the Internet, digital finance boosts the greenization and sustainable development of agriculture (38, 54). Digital finance, as an enabler and conduit for green finance, shares many features with green finance (13). It has been demonstrated that green finance can aid in the greenization of industrial structures (55). Pollution can also be reduced by applying green financial tools, such as green credit and green investment (36, 56, 57). Moreover, industrial structure upgrading can reduce air pollution, thereby improving air quality (58, 59). In summary, we develop the third hypothesis:

H3: Digital finance may curb haze pollution by industrial upgrading effect.

Third, digital finance may contribute to haze pollution reduction by promoting green and sustainable development. At the micro level, digital finance increases borrower and lender transparency, reduces financial risks and borrowing costs, thereby increasing the efficiency of the banking industry (13). All these provide stable financial support for the development of green enterprises, and improve the green total factor productivity of enterprises (28). Muganyi et al. (40) suggested that fintech can address the funding gap of green technologies by improving the enterprises' financial environment, which in turn raises green total factor productivity and reduces energy consumption and pollutant emissions. Besides, at the macro level, digital finance can raise green total factor productivity by improving the agglomeration degree of producer services (25, 56). Zhou et al. (60) discovered that while both fintech and green finance contribute significantly to green growth, there is significant regional heterogeneity in the impact of fintech and green finance on green growth. Some studies have examined the impact of the Internet and financial development on green total factor productivity, with the conclusion that both can contribute significantly to green growth (20, 28). Digital finance, as a new part of the financial system, is bound to influence green growth and air pollution, as well, so we propose the last hypothesis.

H4: Digital finance helps achieve haze pollution reduction through green development effect.

## Institutional background

Back in 1997, China Merchants Bank officially opened its own website and started to provide web technology services for financial institutions. In 2003, Taobao launched Alipay in order to solve the problem of a single form of payment and information asymmetry in e-commerce. With the birth of Alipay, a third-party payment platform, China's digital finance has gradually progressed from technical to commercial. In 2011, the People's Bank of China gave official third-party payment licenses to some third-party platforms, like Alipay, Tenpay, and UnionPay, which created momentum for digital financial development and mobile payments in China.

In 2013, digital finance in China exploded and quickly took the country by storm. The implementation of a series of measures made this year in China the 1st year of digital finance (14, 32, 33). Alipay launched Yu'E Bao in June, which made it possible for digital finance to spread rapidly in China. In the following months, Sina and Tencent launched Weibank and Wechat pay to enter the wealth management market, respectively. Subsequently, Jingdong and NetEase each launched their own financial platforms. Meanwhile, the quick advancement of digital finance is dependent on the support of the Chinese government. In November 2013, the proposal of “developing digital inclusive finance” was officially brought up for the first time at the Third Plenary Session of the 18th Central Committee of the Communist Party of China.

**Figure 1:**
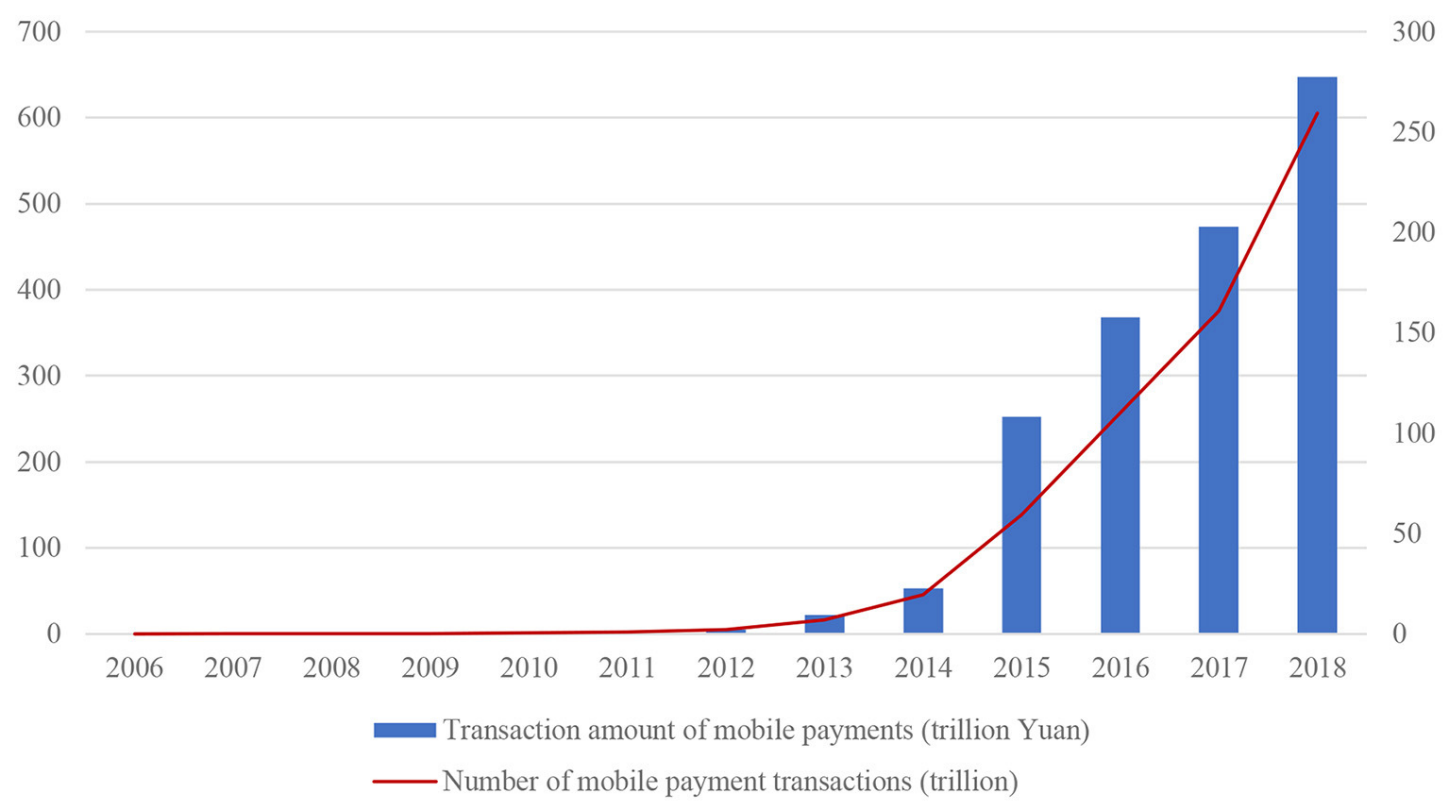
Transaction value and number of transactions of mobile payments in China from 2006 to 2018. Source: The 2019 China Mobile Payment Development Report.

Among all the digital financial tools in China, the mobile payment represented by Alipay is undoubtedly one of the most successful. As shown in Figure 1, after 2013, mobile payments in China grew by leaps and bounds. In addition, according to the 2019 China Mobile Payment Development Report from the State Information Center, over 92% of Chinese mobile payment users use Alipay. As of June 2018, Ant Financial Services Group had partnerships with about 380 Chinese cities and 31 provincial and municipal governments in the areas of digital cities and “Internet+” government services, which cover most aspects of social life. For example, Hainan Province has promoted Alipay's province-wide coverage, Hangzhou buses have all supported Alipay, and citizens in Fuzhou can enjoy convenient services in areas such as life payment and medical care through Alipay. Currently, Alipay is used in a number of economic and social sectors, including those related to transportation, education, health care, dining out, and retail.

**Figure 2:**
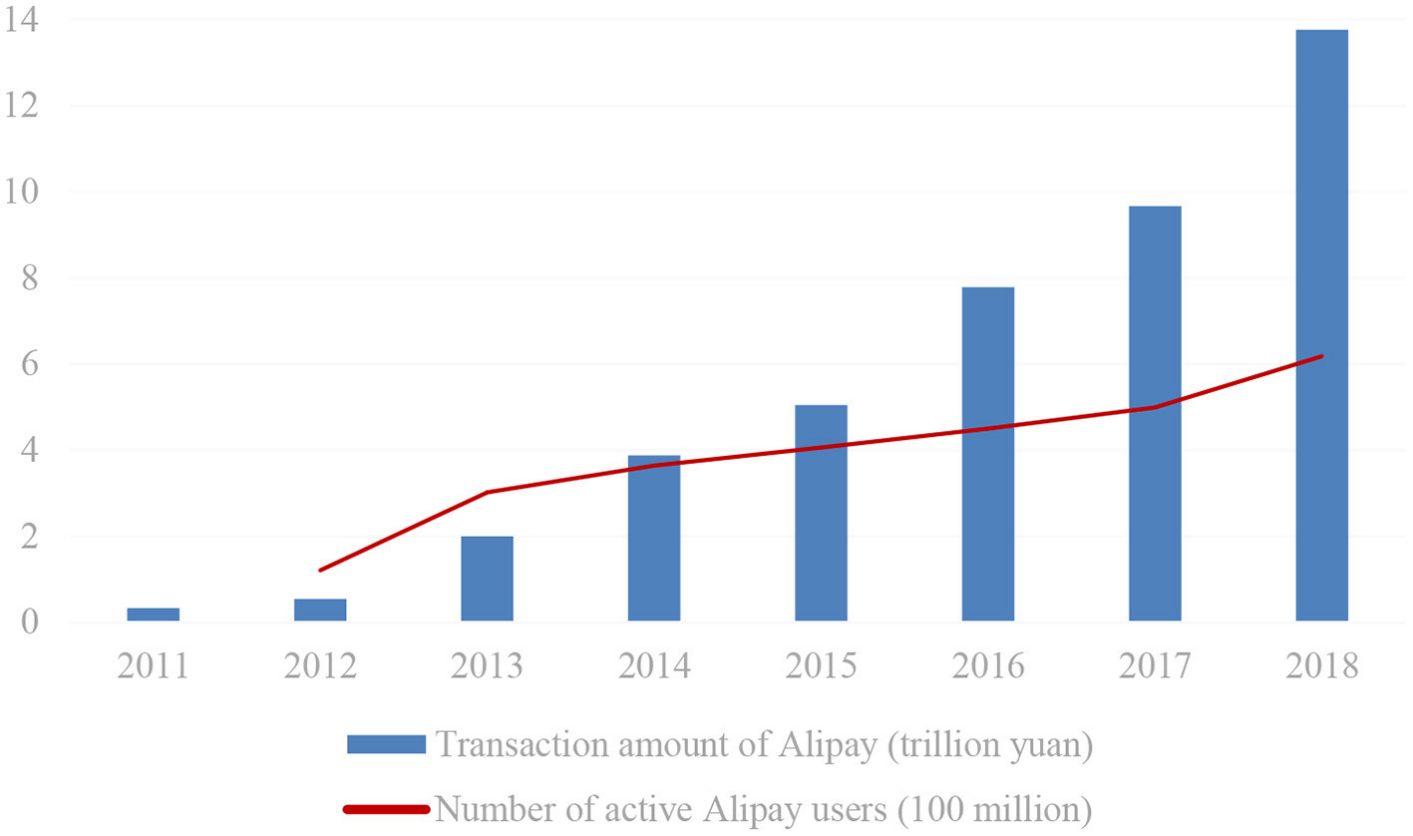
Transaction amount and active users of Alipay from 2011 to 2018. Source: EnfoDesk and Alipay Annual Report.

**Figure 3:**
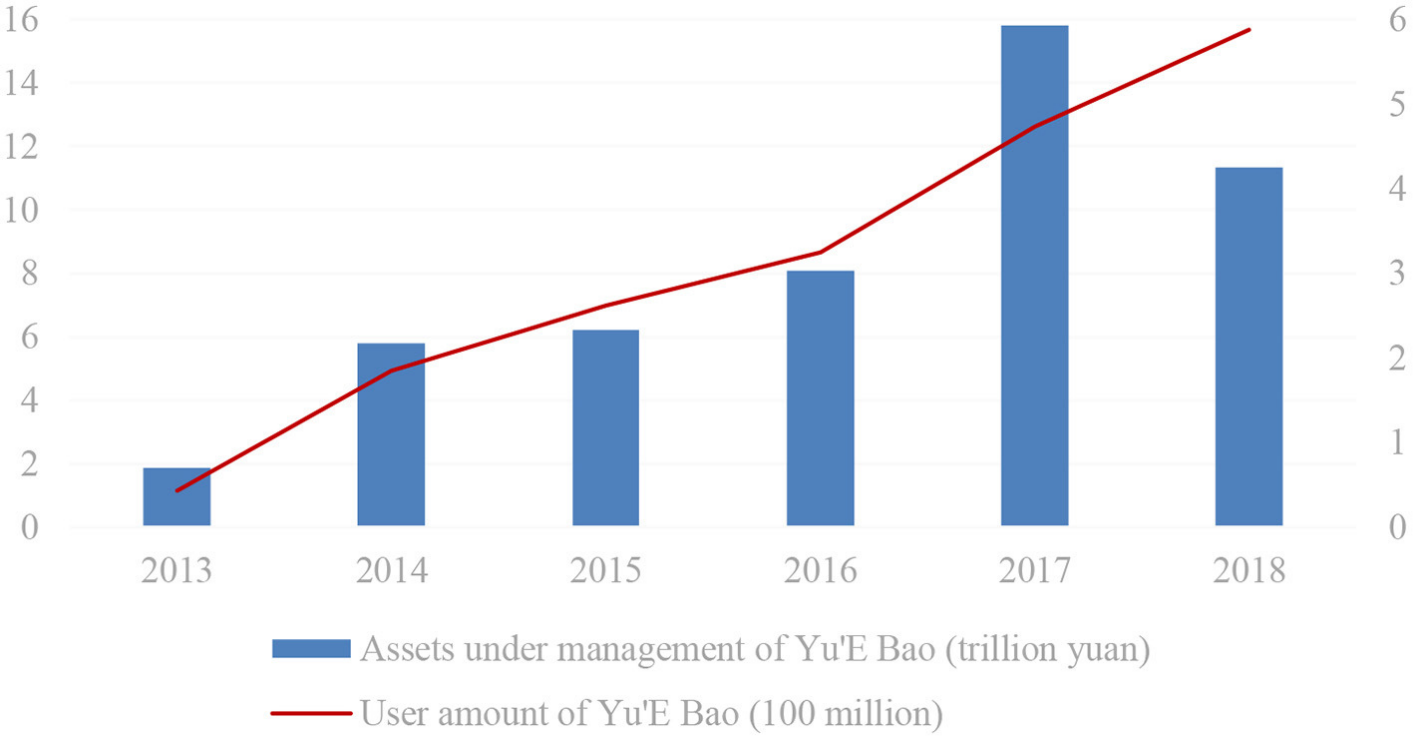
Total assets and users of Yu'E Bao from 2013 to 2018. Source: https://www.statista.com/statistics/1060702/china-mobile-payment-transaction-value/#statisticContainer.

Considering that the digital financial index we used in the later analysis, which is also constructed based on Alipay data, we further describe the situation of Alipay and Yu'E Bao. In Figure 2, it is clear that the total transaction amounts of Alipay rose from 0.34 trillion in 2011 to 13.76 trillion in 2018, including a 268.52% year-on-year increase in 2013. Meanwhile, the active users of Alipay also surpassed 300 million in 2013, 2.50 times more than in 2012. In addition, as shown in Figure 3, since its establishment in June 2013, the number of users of Yu'E Bao has always been growing, and the total size has also maintained a positive trend until 2017. Yu'E Bao, the world's largest money fund, has established a broad market for Alipay and laid the foundation for China's digital financial development.

## Methodology and variables

### Empirical strategy

This paper aims to figure out if there is a causal relationship between digital financial development and haze pollution by using the 1st year of digital finance in China, 2013, as an exogenous shock. The reasons are as follows: First, since 2013, many digital financial tools, such as mobile payment, Internet wealth management, and Internet lending, have rapidly gained popularity in China. Among them, Alipay is the most successful digital financial tool in China, thanks to the launch of Yu'E Bao in June 2013 and the rise of e-commerce (61, 62). As described in Section 3, after 2013, Alipay users and transaction amounts grew rapidly. Second, consistent with the previous studies (10, 11, 15, 19, 20), digital finance is measured by the DFI, which is jointly compiled by the Institute of Digital Finance, Peking University, and Ant Financial. Apparently, the initial data on DFI came from Alipay. Thus, the data and events described above set the stage for us to use the DID model to identify causal relationships. Third, Wang (32) and Zhao et al. (33) also used the exogenous shock of digital financial development in 2013 as a basis for identification. Taken together, the baseline DID model in this study is written as:


(1) $$\begin{array}{r} {PM25}_{it}=a_{0}+a_{1}DFI_{it}\;*\;{Post13}_{t}+a_{2}X_{it}+\delta_{i}+\lambda_{t}+\theta_{pt}+\varepsilon_{it} \end{array}$$

where the subscripts *i, t*, and *p*, respectively, represent the city, year, and province. δ_*i*_, λ_*t*_, and θ_*pt*_ refer to the city fixed effect (FE), year FE, and province-by-year FE, respectively. Controlling for the high-dimensional FE mitigates the potential impact of certain characteristics that vary from year to year at the provincial level on our estimates (33, 63). *PM*25_*it*_ denotes the haze pollution of city *i* in year *t*, which is widely used in previous studies (2, 64, 65). *DFI*_*it*_ is a continuous variable indicating the development status of digital finance in city *i* in year *t*; *Post*13_*t*_ is a dummy variable, which equals to 1 if the year is in 2013 and later, and 0 otherwise. The coefficient *a*_1_ on the interaction item *DFI*_*it*_ * *Post*13_*t*_ is the DID estimator we are concerned about. *X*_*it*_ refers to a set of control variables, which includes city characteristics and climate characteristics. ε_*it*_ is the random error term.

Note that the difference between the model we set up and the traditional DID model is that DFI is a continuous variable in equation (1), which is because we cannot strictly distinguish between control and treated groups. In fact, this approach is often used in DID models, and also yields a net effect under causal identification (66, 67). For example, in the study of Chen et al. (66), the number of educated youth in each county was used to reflect the degree of the Send-Down Movement, and Chen and Zhou (68) employed excess mortality as a proxy for famine shocks. More importantly, exploiting continuous variables in the DID framework can better capture the extent of exposure to policy influence, which is increasingly accepted by scholars and is gaining popularity. In this paper, we do not set a dummy variable to construct a traditional DID model based on the median or mean of DFI, because it is impossible to avoid additional endogeneity problems from human intervention. Therefore, cities with relatively high levels of digital finance are regarded as potential treated groups, while cities with relatively low levels of digital finance are considered potential control groups.

Additionally, the parallel trend assumption must be satisfied to use the DID model. In other words, before exogenous shocks to digital finance occurred in 2013, the haze pollution status of the cities needed to have the same trend. Drawing on the event study approach (66, 69, 70), we utilize the following model to conduct the parallel trend tests.


(2) $$\begin{array}{l} \begin{array}{ccc} PM25_{it} & = & \beta_{0}+\sum^{\text{​}}\beta_{\tau }DFI_{i\tau }*Post13_{\tau }+\beta_{2}X_{it}+\delta_{i} \end{array}\\ \;\begin{array}{cc} + & \lambda_{t}+\theta_{pt}+\mu_{it} \end{array} \end{array}$$

where *DFI*_*iτ*_ * *Post*13_τ_ represents the relative year τ of city *i* from being influenced by the exogenous shock for digital finance in 2013. τ equals to 0 indicates the year in which the exogenous shock occurred. Thus, a negative (positive) τ indicates before (after) the exogenous shock. For example, τ equals to−1 is the 1st year prior to the exogenous shock. If the coefficient β_τ_ is not significant when τ is negative, it means that the parallel trend is satisfied.

### Variables

#### Haze pollution

Haze pollution is our explained variable, which is measured by the annual average concentration of urban PM_2.5_ in this paper. This is because PM_2.5_ is the main source of haze pollution in Chinese cities (4, 7, 64). Compared with other air pollutants, such as PM_10_, PM_2.5_ is more destructive to the respiratory and immune systems, and is more likely to cause respiratory and cardiovascular diseases (4, 71). The Chinese government and the public have currently focused on haze pollution caused by PM_2.5_ (2). The PM_2.5_ concentration data for each city in this study is from the Atmospheric Composition Analysis Group at Washington University in St. Louis^5^.

#### Digital finance

**Figure 4:**
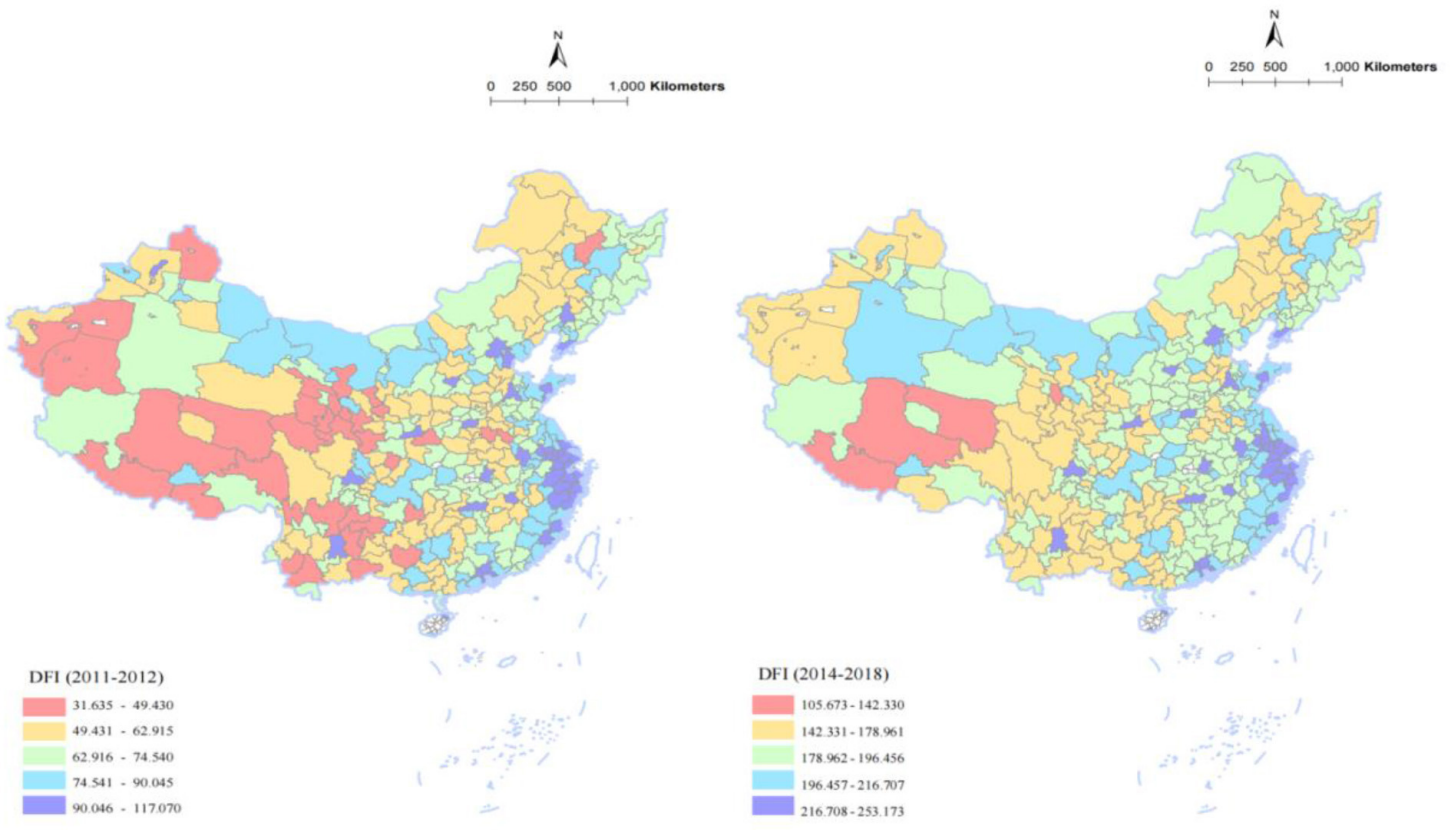
Digital finance level in Chinese cities (2011–2018).

As mentioned in the above sections, digital finance is measured by the DFI, published by the Institute of Digital Finance, Peking University. This index records the digital financial development in China in detail, covering three levels: province, prefecture-level city, and county. The index also includes three secondary indicators: coverage breadth (CBI), usage depth (UDI), and digitization level (DLI). In Figure 4, we draw the geographical distribution map of prefecture-level digital finance around 2013. On average, China's digital finance has tripled since 2013. In addition, some cities with relatively developed economies have higher levels of digital finance, such as the Yangtze River Delta, the Pearl River Delta, and the Beijing-Tianjin-Hebei city clusters.

#### Control variables

To accurately capture the impact of digital financial development on haze pollution and reduce the endogeneity, we must introduce some control variables. Referring to the previous literature (2, 7, 29, 30, 72), city characteristics and climate characteristics are two levels of control variables in our study. First, for city characteristics variables, considering the environmental Kuznets curve (20), we control for *GDP per capita, Fiscal expenditures*, and *Fiscal revenue*. Human capital, as measured by *Education expenditures* and *University*, is also included as a control variable (2, 43). In addition, we consider *Transport infrastructure* and *Savings*, due to the potential impact of infrastructure, savings, and consumption on haze pollution (7, 30). Moreover, given that some previous scholars found that the environmental impact of foreign investment and industrial enterprises (29, 72), *FDI, Foreign-owned enterprises*, and *Above-scale enterprises* are taken into account. Finally, in particular, we control for *Internet penetration rate, Financial development*, and *Postal services* that are strongly tied to the development of digital finance and may affect haze pollution (37, 38). Adding them to our regressions helps to overcome the potential endogeneity problem.

Second, similar to prior research (69, 70), climate characteristics cover three variables, *Temperature, Relative humidity*, and *Precipitation*, as the influence of climatic factors on haze pollution is gradually attracting attention. The definitions of above variables are listed in Table 1. The data on city and climate characteristics comes from the China City Statistical Yearbook and the National Weather Service, respectively.

**Table 1:** Variable definitions and descriptive statistics.

**Variables**	**Definition**	***N***	**Mean**	**SD**
PM_2.5_	Annual average concentration of PM_2.5_ (μg/m^3^)	1921	43.5086	19.280
DFI	Digital Finance Index for prefecture-level cities	1921	152.188	60.628
CBI	Coverage breadth of digital finance	1921	143.069	58.210
UDI	Usage depth of digital finance	1921	150.583	63.310
DLI	Digitization level of digital finance	1921	185.678	80.704
Post13	= 1 if the year is 2013 and later; 0 otherwise	1921	0.7361	0.4409
DFI*Post13	The interaction item of DFI and post13	1921	133.056	87.305
GDP per capita	GDP per capita of the city (10,000 yuan)	1921	5.0261	3.0109
Fiscal expenditures	The radio of fiscal expenditures to GDP	1921	0.1855	0.0797
Fiscal revenue	The radio of fiscal revenue to GDP	1921	0.0786	0.0266
Education expenditures	The radio of education expenditures to the total fiscal expenditures	1921	0.1801	0.0388
University	Number of universities or colleges	1921	1.7509	0.8981
Transport infrastructure	Road haulage traffic (100 million tons)	1921	1.1842	0.9640
Savings	Total savings in cities (100 million yuan)	1921	3.7081	2.7790
FDI	The radio of amount of foreign capital actually utilized to the GDP (multiplied by 100)	1921	1.6777	1.5340
Foreign-owned enterprises	Number of foreign-owned enterprises in the city (logarithmic)	1921	3.2059	1.4621
Above-scale enterprises	Number of above-scale enterprises in the city (logarithmic)	1921	6.6838	0.9976
Temperature	Annual average temperature of the city (°C)	1921	15.2703	4.9668
Relative humidity	Annual relative humidity of the city	1921	0.6890	0.0934
Precipitation	Annual precipitation of the city (mm/100)	1921	0.4057	3.0794
Internet penetration rate	The number of international Internet users to the total population	1921	0.2036	0.1586
Financial development	Total bank loans and deposits divided by the GDP	1921	2.3060	1.0688
Postal services	Total postal services of the city (10,000 yuan, logarithmic)	1921	10.5842	1.1074

### Descriptive statistics

The descriptive statistics of the variables are shown in Table 1, with all continuous variables being winsorized to remove the effect of extreme values. We can see that the annual average PM_2.5_ concentrations are 43.5086 μg/m^3^, indicating that pollution in Chinese cities is generally severe, according to the World Health Organization's classification of the annual average concentrations of PM_2.5_. In addition, the mean of DFI at the prefecture-city level is 152.188; the mean values of the three sub-indicators of DFI, namely CBI, UDI, and DLI, are 143.069, 150.583, and 185.678, respectively. Moreover, for control variables, the average GDP per capita is 50.261 thousand Yuan, and the mean ratios of fiscal expenditure and fiscal revenue to GDP are 18.55 and 7.86%, respectively. The average Internet penetration rate and traditional financial development index are 20.36% and 2.3060.

## Results

### Baseline results

According to model (1), the DID method is used to look into the effect of digital finance on haze pollution. The baseline results are represented in Table 2, with all standard errors clustered at the prefecture-city level. Considering the potential sensitivity of estimates for the core explanatory variable to controls, we added these control variables in batches in each column. Specifically, in column (1), we control for some basic city characteristics variables and then further control for climate variables in column (2). As can be seen, both DFI^*^Post13 coefficients are significantly negative. In column (3), we further include three important control variables, *Internet penetration rate, Financial development*, and *Postal services*. It is obvious that the DID estimate remains significantly negative. Finally, in column (4), we control for the province-by-year FE, a set of high-dimensional FE, finding that the *DFI* * *Post13* coefficient is also significantly negative at the 1% level.

**Table 2:** The effects of digital finance on air pollution: DID methods.

	**(1)**	**(2)**	**(3)**	**(4)**
DFI*Post13	−0.0462***	−0.0459***	−0.0417***	−0.0598***
	(0.0140)	(0.0142)	(0.0152)	(0.0189)
GDP per capita	−0.4753**	−0.4628**	−0.3967*	0.2401**
	(0.2075)	(0.2068)	(0.2080)	(0.1152)
Fiscal expenditures	24.1045*	23.6827*	21.0556	−2.7074
	(13.1553)	(13.1445)	(13.1727)	(5.3868)
Fiscal revenue	−85.8466***	−84.7290***	−83.8340***	−24.2515**
	(18.6175)	(18.4069)	(18.0406)	(10.3356)
Education expenditures	4.7590	4.3414	3.5617	5.0075
	(10.5647)	(10.6020)	(10.6283)	(6.9693)
University	−0.7368	−0.7970	−0.8414	1.5586**
	(1.4179)	(1.4209)	(1.4074)	(0.7594)
Transport infrastructure	0.1004	0.1114	0.0971	0.0560
	(0.4500)	(0.4503)	(0.4508)	(0.2378)
Savings	0.8375**	0.8065**	0.8052**	−0.5251
	(0.3736)	(0.3788)	(0.3824)	(0.3479)
FDI	−0.6067**	−0.6061**	−0.6039**	−0.0111
	(0.2476)	(0.2455)	(0.2431)	(0.1202)
Foreign-owned enterprises	−0.9952	−1.0096	−1.0421	−0.1829
	(0.8997)	(0.9136)	(0.9310)	(0.5595)
Above-scale enterprises	−0.6905	−0.5491	−0.1778	−0.4216
	(1.1214)	(1.1242)	(1.1755)	(0.9620)
Temperature		0.0106	0.0644	−0.6961
		(0.4799)	(0.4803)	(0.4766)
Relative humidity		−5.5241	−4.4828	−1.9996
		(6.2296)	(6.2740)	(4.4658)
Precipitation		−0.0340	−0.0297	−0.0259
		(0.0628)	(0.0632)	(0.0405)
Internet penetration rate			−2.3942	−3.8272***
			(2.5325)	(1.4680)
Financial development			0.7095	−0.0464
			(0.7070)	(0.4002)
Postal services			−0.3988	−0.0938
			(0.3972)	(0.2083)
City FE	Yes	Yes	Yes	Yes
Year FE	Yes	Yes	Yes	Yes
Province-by-year FE	No	No	No	Yes
Adjusted *R*^2^	0.9344	0.9343	0.9345	0.9769
*N*	1921	1921	1921	1921

*, **, *** represent the significant levels of 10%, 5%, and 1%, respectively. Parentheses indicate standard errors clustered at the city level.

Not only statistically, but also economically, this paper finds that digital finance has a substantial effect on haze pollution. In particular, in column (4), our preferred specification, the DFI^*^Post13 coefficient is−0.0598, indicating that after 2013, with rapid growth in digital finance, a one-unit increase in the digital financial index resulted in an average decrease of 5.98% in PM_2.5_ concentrations in Chinese cities. The results show that a one-standard deviation increase in digital finance after 2013 decreases the PM_2.5_ concentrations by 0.2708 standard deviations when converted using standard deviations. Taken together, these findings support the **H1** that digital financial development does mitigate haze pollution in China.

In combined above, the Internet penetration rate is negatively correlated with haze pollution. However, the relationship between GDP per capita and haze pollution is not stable, which may be influenced by the environmental Kuznets curve (20). In addition, fiscal revenue is negatively related to haze pollution, which may be influenced by the environmental control and environmental protection policies of local governments (30, 64).

### Parallel trend tests

**Figure 5:**
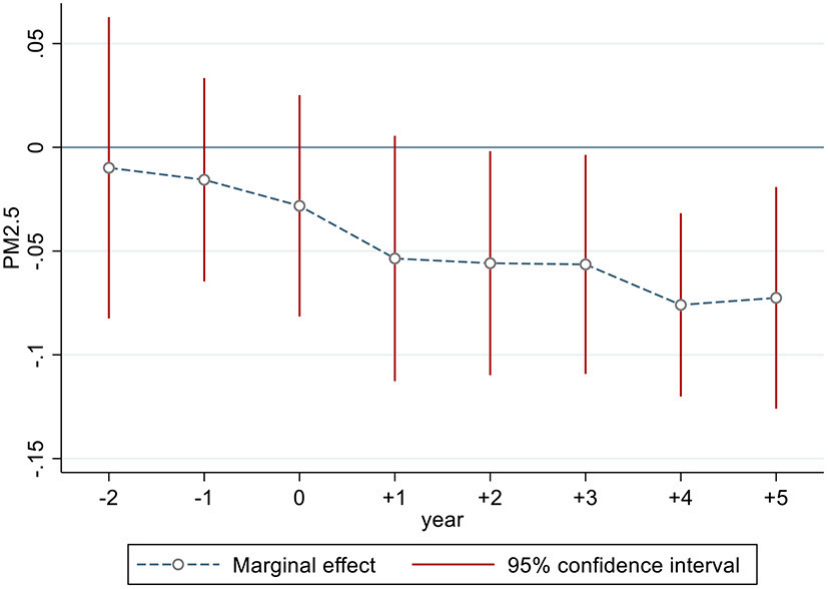
Parallel trend tests.

As discussed in Section Methodology and variables, the crucial prerequisite for adopting the DID method is to satisfy the parallel trend tests. The estimated results based on model (2) are depicted in Figure 5 by using the event study. The estimated coefficients are both insignificant at the 5% level^6^ before period 0 (in 2013), indicating that the parallel trend is satisfied, and our DID estimates in Table 2 are quite convincing. After period +2, the estimated coefficients become significantly negative at the 5% level. Moreover, we find the coefficients decrease over time, implying that the marginal effect of digital finance development on reducing PM_2.5_ concentrations gradually increases. Overall, these results further suggest that digital financial development is responsible for haze pollution reduction in Chinese cities.

### Mechanism tests

To further test the subsequent hypotheses, we examine three mechanisms for digital finance to reduce haze pollution: technological innovation, industrial upgrading, and green development. Due to the availability of data for the mechanism variables, the sample size of some regressions may be different from the main results.

#### Technological innovation

We consider whether digital finance affects technological innovation, as highlighted in **H2**. City technological innovation is measured by patents, as the core innovation output (17, 73). In line with previous studies, the original data for the patents came from the China National Intellectual Property Administration (2, 45). More importantly, this patent database contains information on green patents, including two types: green inventions and green utility models. Thus, we first construct five mechanism variables: *Patents granted, Green invention patents applications, Green invention patents granted, Green utility model patents applications*, and *Green utility model patents granted*. The latter four variables are more reflective of a city's green innovation capacity (For detailed descriptions, see the Panel A of Table A1 in the Appendix). The first five columns of Table 3 imply that digital financial development only promotes green patents, while the effects on total patents granted and green utility model patents are not statistically significant. These results are consistent with prior literature (10, 11). Considering the role of technological innovation, especially green innovation, in reducing air pollution (6), we consider that digital finance has the potential to reduce haze pollution by encouraging green innovations in cities.

**Table 3:** Mechanism tests: Digital finance and technology innovation.

	**(1)**	**(2)**	**(3)**	**(4)**	**(5)**	**(6)**	**(7)**
	**Patents granted**	**Green patents applications**	**Green patents granted**	**Green utility model patents applications**	**Green utility model patents granted**	**City Innovation Power Index**	**City Innovation and Entrepreneurship Index**
DFI*Post13	0.0007	0.0033**	0.0037***	−0.0017	0.0008	0.0052**	0.0003
	(0.0007)	(0.0016)	(0.0011)	(0.0011)	(0.0012)	(0.0021)	(0.0002)
Baseline controls	Yes	Yes	Yes	Yes	Yes	Yes	Yes
City FE	Yes	Yes	Yes	Yes	Yes	Yes	Yes
Year FE	Yes	Yes	Yes	Yes	Yes	Yes	Yes
Province-by-year FE	Yes	Yes	Yes	Yes	Yes	Yes	Yes
Adjusted *R*^2^	0.9427	0.9604	0.9621	0.9647	0.9637	0.8931	0.9583
*N*	1921	1471	1469	1471	1469	1472	1921

**, *** represent the significant levels of 5%, and 1%, respectively. Parentheses indicate standard errors clustered at the city level. The baseline control variables are consistent with column (4) in Table 2.

In addition to patents, there are also some comprehensive indicators that can reflect cities' overall innovation capacity, represented by the City Innovation Power Index and the City Innovation and Entrepreneurship Index (54, 69)^7^ (See Panel A of Table A1 in the Appendix for details). As shown in column (6) of Table 3, the coefficient of DFI^*^Post13 is significantly positive, which indicates that the Chinese cities' innovation capability is considerably increased by digital financial development. Therefore, the results in Table 3 confirm that digital finance does play a role in city innovation and back up our **H2**.

#### Industrial upgrading

As discussed in Section Literature review and hypothesis development, another important channel for haze pollution reduction is industrial upgrading. Referring to the previous studies [e.g., Chen and Zhang (16); Zhao et al. (17); Han et al. (58)], we select four variables to characterize city industrial upgrading: *Industrial structure index, Ratio of tertiary industry to secondary industry, Tertiary industry efficiency*, and *Manufacturing*. The first two reflect the optimization and upgrading of the overall industrial structure in cities (17); the third describes the development efficiency of the tertiary sector, which has a relatively small negative impact on haze pollution; the last reflects the internal structure of the secondary sector, as manufacturing is one of the primary producers of air pollutants in China (74) (see Panel B of Table A1 in the Appendix).

Except for column (3), all the estimated coefficients of DFI^*^Post13 are significantly positive in Table 4, which indicates that the development of digital finance leads to urban industrial upgrading. Specifically, digital finance leads to an increase of 0.08 points in the industrial structure index, promotes employment in the tertiary sector relative to the secondary sector, and decreases manufacturing employment. However, digital finance has failed to improve tertiary industry efficiency. Although digital finance brings new impetus for the tertiary sector (11, 51), efficiency improvement requires long-term accumulations of human capital, physical capital, and technology (75). Current digital financial development is insufficient to support the efficient growth of the tertiary industry. These results back up our **H3** and show that digital financial development helps with industrial upgrading, which could help cut down on haze pollution even more (58, 59).

**Table 4:** Mechanism tests: Digital finance and industrial upgrading.

	**(1)**	**(2)**	**(3)**	**(4)**
	**Industrial structure index**	**Ratio of tertiary industry to secondary industry**	**Tertiary industry efficiency**	**Manufacturing**
DFI*Post13	0.0007***	0.0075***	−0.0003	−0.0010***
	(0.0003)	(0.0018)	(0.0003)	(0.0003)
Baseline controls	Yes	Yes	Yes	Yes
City FE	Yes	Yes	Yes	Yes
Year FE	Yes	Yes	Yes	Yes
Province-by-year FE	Yes	Yes	Yes	Yes
Adjusted R^2^	0.9009	0.8859	0.8533	0.9102
N	1883	1919	1670	1878

*** represent the significant levels of 1%. Parentheses indicate standard errors clustered at the city level. The baseline control variables are consistent with column (4) in Table 2.

#### Green development

As emphasized in **H4**, the last potential explanation for the haze pollution reduction in the digital finance era is green development. Similar to most of the previous literature, we use green total factor production (GTFP) to represent the green development of cities [e.g., Liu et al. (24); Zhou et al. (60); Xia and Xu (76)]. We adopt the Malmquist-Luenbergen Index to calculate the GTFP. Physical capital, labor, land, and energy are considered the input indicators. The expected output is GDP, while non-expected output includes industrial wastewater discharges, industrial sulfur dioxide emissions, and industrial soot emissions. These indicators' descriptions are shown in Table A2 of the Appendix, and the China City Statistical Yearbook provides their original data. Furthermore, the GTFP can be divided into two categories: efficiency change (EC) and technology change (TC) (see Panel C of Table A1 in the Appendix).

In column (1) of Table 5, the DID estimate shows that the coefficient of DFI^*^Post13 is significantly positive at the 5% level, revealing that digital financial development increases the GTFP and promotes green development in Chinese cities. This finding is consistent with previous studies (24, 56). Considering the close positive relationship between the green development model and haze pollution reduction (28, 77), we confirm the reliability of **H4** that digital financial development can help reduce PM_2.5_ concentrations by promoting green and sustainable development. Moreover, in columns (2) and (3), for two sub-indicators, we find that digital finance considerably contributes to technology change but does not have a significant impact on efficiency change. There are a lot of similarities between these estimates and the previous one that examined the impact of digital finance on innovation capability in Chinese cities.

**Table 5:** Mechanism tests: Digital finance and green development.

	**(1)**	**(2)**	**(3)**
	**GTFP**	**EC**	**TC**
DFI*Post13	0.0012**	0.0001	0.0014***
	(0.0006)	(0.0007)	(0.0005)
Baseline controls	Yes	Yes	Yes
City FE	Yes	Yes	Yes
Year FE	Yes	Yes	Yes
Province-by-year FE	Yes	Yes	Yes
Adjusted *R*^2^	0.5723	0.0723	0.6228
*N*	1219	1219	1219

**, *** represent the significant levels of 5%, and 1%, respectively. Parentheses indicate standard errors clustered at the city level. The baseline control variables are consistent with column (4) in Table 2.

### Heterogeneity effects

In this subsection, we explore the potential heterogeneity effect in several ways. First, as mentioned in Section 4, the digital finance index includes three secondary indicators, CBI, UDI, and DLI. In the framework of the DID model, we construct three interaction terms, CBI^*^Post13, UDI^*^Post13, and DLI^*^Post13. The coefficients of the interaction terms are significantly negative in columns (1) and (2) of Table 6, demonstrating that the coverage breadth and usage depth of digital finance contribute to haze pollution mitigation. More specifically, these estimates suggest that after 2013, the PM_2.5_ concentrations decreased by 0.0993 and 0.1789 standard deviations for each standard deviation increase in coverage breadth and usage depth, respectively. Therefore, in comparison, the usage depth of digital finance may help lessen haze pollution in China more.

**Table 6:** Heterogeneity effects for sub-indexes of digital finance.

	**(1)**	**(2)**	**(3)**
CBI*Post13	−0.0329**		
	(0.0131)		
UDI*Post13		−0.0545***	
		(0.0161)	
DLI*Post13			−0.0074
			(0.0052)
Baseline controls	Yes	Yes	Yes
City FE	Yes	Yes	Yes
Year FE	Yes	Yes	Yes
Province-by-year FE	Yes	Yes	Yes
Adjusted *R*^2^	0.9767	0.9771	0.9765
*N*	1921	1921	1921

**, *** represent the significant levels of 5%, and 1%, respectively. Parentheses indicate standard errors clustered at the city level. The baseline control variables are consistent with column (4) in Table 2.

Second, we performed some tests of heterogeneity at the city level. In terms of geographical location, northern Chinese cities have experienced more severe haze pollution in recent years (59, 64). Therefore, using the Qinling-Huaihe Line as the dividing line, the Chinese cities are split into two sub-samples: those in the north and those in the south (2, 17, 69). In the first two columns of Table 7, the coefficient of DFI^*^Post13 is only significant in the column (1), suggesting that digital finance can produce greater positive benefits in the northern regions where haze pollution is more severe. Moreover, considering the close correlation between digital finance and the Internet foundation and financial development (13, 15, 17), digital finance is likely to grow faster in regions with higher levels of Internet penetration and financial development. Thus, the cities are divided into two sub-samples based on the medians of *Internet penetration rate* and *Financial development*. The last four columns of Table 7 show that digital finance significantly cuts haze pollution by a lot in cities that have better levels of Internet foundation and traditional financial development. In other words, a better information technology base and traditional financial base create better conditions for digital finance to curb haze pollution.

**Table 7:** Heterogeneity effects at the city level.

	**(1)**	**(2)**	**(3)**	**(4)**	**(5)**	**(6)**
	**Northern cities**	**Southern cities**	**Better internet foundation**	**Worse internet foundation**	**Higher financial development level**	**Lower financial development level**
DFI*Post13	−0.1182***	0.0152	−0.1112***	−0.0030	−0.1209***	0.0036
	(0.0281)	(0.0180)	(0.0335)	(0.0250)	(0.0251)	(0.0243)
Baseline controls	Yes	Yes	Yes	Yes	Yes	Yes
City FE	Yes	Yes	Yes	Yes	Yes	Yes
Year FE	Yes	Yes	Yes	Yes	Yes	Yes
Province-by-year FE	Yes	Yes	Yes	Yes	Yes	Yes
Adjusted *R*^2^	0.9786	0.9755	0.9757	0.9848	0.9725	0.9808
*N*	827	1094	960	961	960	961

*** represent the significant levels of 1%. Parentheses indicate standard errors clustered at the city level. The baseline control variables are consistent with column (4) in Table 2.

Third, given that the positive effects of digital finance may differ at different PM_2.5_ concentrations, we conduct quantile regressions for further heterogeneity analysis (70, 78). Table 8 shows that estimated coefficients on DFI^*^Post13 are significantly negative in columns (2), (3), and (4), but insignificant in columns (1) and (5). These results imply that digital finance has a more significant impact in the middle of the conditional distribution for haze pollution than at either end. That is, the impact of digital financial development on cities with very light or very severe haze pollution is insignificant. Digital finance is a non-mandatory approach to haze pollution control, therefore, for some cities with very serious air pollution, the effect may be limited. In contrast, cities in China that are barely affected by haze pollution are mainly dominated by tourism and tertiary industries and are relatively less dependent on digital finance. Moreover, digital financial instruments do not contribute to further improving the marginal environmental benefits of these low-pollution cities.

**Table 8:** Quantile regressions.

	**(1)**	**(2)**	**(3)**	**(4)**	**(5)**
	**QR_10**	**QR_30**	**QR_50**	**QR_70**	**QR_90**
DFI*Post13	−0.0282	−0.0531***	−0.0670***	−0.0579***	−0.0175
	(0.0224)	(0.0139)	(0.0130)	(0.0128)	(0.0165)
Baseline controls	Yes	Yes	Yes	Yes	Yes
City FE	Yes	Yes	Yes	Yes	Yes
Year FE	Yes	Yes	Yes	Yes	Yes
Province-by-year FE	Yes	Yes	Yes	Yes	Yes
*N*	1921	1921	1921	1921	1921

*** represent the significant levels of 1%. Parentheses indicate standard errors clustered at the city level. The baseline control variables are consistent with column (4) in Table 2.

### Robustness checks

To confirm the reliability of our baseline results in Table 2, we perform several robustness tests, including placebo tests, controlling for disruptive policies, IV estimations, using alternative specifications, and adopting the county-level data.

#### Placebo tests

2013 was the 1st year of digital finance in China. It can be used as an exogenous shock to determine the causal relationship between digital financial development and haze pollution. In the placebo tests, we advance this shock by 1 year to 2012. The first column of Table 9 shows that the estimated coefficient on DFI^*^Post12 is insignificant, Implying that 2013 is valid as an exogenous shock to digital financial development in China. In addition, as discussed in Section 4, although *DFI* is a continuous variable, in the DID specification, cities with better digital financial development are considered a potential treated group, while cities with low levels of digital finance are regarded as a potential control group (66, 67). Therefore, two pseudo-treated groups are constructed. On the one hand, we define cities with negative digital finance growth as the pseudo-treated group I. On the other hand, we consider cities with digital finance in the bottom 25% of the quantile as the pseudo-treated group II. Clearly, in Table 9 columns (2) and (3), the coefficients on *DFI*^pseudo^*Post13 are both insignificant. In sum, these placebo tests further strengthen the persuasiveness of our baseline results.

**Table 9:** Placebo tests.

	**(1)**	**(2)**	**(3)**
	**One year in advance**	**Pseudo-treated group I**	**Pseudo-treated group II**
DFI*Post12	−0.0187		
	(0.0261)		
DFI^pseudo^*Post13		−0.0025	−0.0404
		(0.0045)	(0.0277)
Baseline controls	Yes	Yes	Yes
City FE	Yes	Yes	Yes
Year FE	Yes	Yes	Yes
Province-by-year FE	Yes	Yes	Yes
Adjusted *R*^2^	0.9774	0.9764	0.9765
*N*	1921	1921	1921

Parentheses indicate standard errors clustered at the city level. The baseline control variables are consistent with column (4) in Table 2.

#### Disruptive policies

During the study period, there are some policies related to digital finance, including Smart Cities, Low-carbon Cities, Forest Cities, APPCAP, and Broadband China, that may affect our estimations. Therefore, we further control these policies to perform robustness tests (66, 70). Among them, Smart Cities and Broadband China policies directly improve the Internet infrastructure of cities (79, 80), which may not only affect digital financial development, but also promote green development and reduce PM_2.5_ concentrations (81). The other three belong to environment-related policies, whose effectiveness in improving air pollution has been confirmed by previous studies (64, 69).

We follow the DID specification to construct dummy variables to represent the potential impact of these policies. Specifically, a city is set to 1 when it becomes a pilot city in a given year and 0 otherwise. In the first five columns of Table 10, we control for these policies, respectively, finding that our main results are not sensitive to these disruptive policies. In column (6), after controlling for all five policies, the estimates show that the coefficient on DFI^*^Post13 remains significantly negative.

**Table 10:** Robustness checks by controlling for disruptive policies.

	**(1)**	**(2)**	**(3)**	**(4)**	**(5)**	**(6)**
	**Smart cities**	**Low-carbon cities**	**Forest cities**	**APPCAP**	**Broadband China**	**All policies**
DFI*Post13	−0.0639***	−0.0611***	−0.0598***	−0.0629***	−0.0642***	−0.0707***
	(0.0202)	(0.0191)	(0.0189)	(0.0189)	(0.0192)	(0.0204)
Baseline controls	Yes	Yes	Yes	Yes	Yes	Yes
City FE	Yes	Yes	Yes	Yes	Yes	Yes
Year FE	Yes	Yes	Yes	Yes	Yes	Yes
Province-by-year FE	Yes	Yes	Yes	Yes	Yes	Yes
Adjusted *R*^2^	0.9769	0.9769	0.9769	0.9770	0.9770	0.9771
*N*	1921	1921	1921	1921	1921	1921

*** represent the significant levels of 1%. Parentheses indicate standard errors clustered at the city level. The baseline control variables are consistent with column (4) in Table 2.

#### IV estimations

The IV approach is applied in the paper to further eliminate potential endogeneity. Specifically, we select the interaction term between average digital finance within the same province and *Post13* as our IV. On the one hand, due to the spillover effects of digital finance, the digital finance development in a city is closely related to other cities in the same province (12). Therefore, theoretically, this IV satisfies the correlation assumption. On the other hand, since we strictly control the city FE and province-by-year FE, digital finance in other cities is hard to influence the haze pollution in a certain city through other channels directly. Thus, we believe that this IV also theoretically satisfies the exclusion restriction hypothesis.

The results of IV estimation using the two-stage least squares (2SLS) method are reported in Table 11. There is a significant positive correlation between IV and the core explanatory variable, suggesting that the correlation assumption is satisfied. In addition, the first-stage F value is greater than the criticality of 10, and the Cragg-Donald Wald F statistic and Kleibergen-Paap rk LM statistic are within a reasonable range. These statistics indicate that our estimations exclude the issue of weak IV. Furthermore, in the second stage, the coefficient of DFI^*^Post13 remains significantly negative at the 1% level, indicating that our estimates remain robust after further eliminating potential endogeneity problems after using the IV estimations.

**Table 11:** IV estimations.

	**(1)**	**(2)**
	DFI*Post13	**PM**_**2.5**_
IV*Post13	0.3910***	
	(0.1192)	
DFI*Post13		−0.5127***
		(0.1638)
First-stage F value	10.7570	
Cragg-Donald Wald F statistic		72.0772
Kleibergen-Paap rk LM statistic		3.5614
*P*-value		0.0591
Baseline controls	Yes	Yes
City FE	Yes	Yes
Year FE	Yes	Yes
Province-by-year FE	Yes	Yes
*N*	1921	1921

*** represent the significant levels of 1%. Parentheses indicate standard errors clustered at the city level. The baseline control variables are consistent with column (4) in Table 2.

#### Using alternative specifications

We conduct some robustness checks by using alternative specifications. First, we exclude all municipalities, sub-provincial cities, and provincial capitals, as the inconsistency in administrative status leads to the possibility that these cities may have an advantage in developing digital finance. Second, the impact of haze pollution in cities may be amplified by time trends. This is because digital finance is closely tied to some factors, such as the cities' geographic locations, socioeconomic development, and openness. Thus, employing the method of Lu et al. (82), we include some interaction terms between these factors and linear trends over time in the baseline regression^8^. Third, we add some other control variables, namely *Fixed asset investment, Population density, Economic growth rate*, and *Salary per capita*. Fourth, we construct balanced panel data. Fifth, we extend the years to 2008 to guarantee the consistency of the years before and after the exogenous shock in 2013. Finally, there is a synergistic effect with PM_2.5_ and SO_2_, which is another major air pollutant in Chinese cities (83). Therefore, we use SO_2_ emissions as a proxy for the explained variable. In Table 12, we find the DID estimate of DFI^*^Post13 is unchanged, which indicate that our main results are quite satisfactory in capturing these alternative specifications.

**Table 12:** Robustness checks by using alternative specifications.

	**(1)**	**(2)**	**(3)**	**(4)**	**(5)**	**(6)**
	**Deleting special cities**	**Controlling time trends**	**Adding other controls**	**Balancing panels**	**2008-2018**	**SO_2_ emissions**
DFI*Post13	−0.0851***	−0.0596***	−0.0647***	−0.0596***	−0.0524***	−0.0040***
	(0.0257)	(0.0189)	(0.0215)	(0.0191)	(0.0190)	(0.0011)
Baseline controls	Yes	Yes	Yes	Yes	Yes	Yes
City FE	Yes	Yes	Yes	Yes	Yes	Yes
Year FE	Yes	Yes	Yes	Yes	Yes	Yes
Province-by-year FE	Yes	Yes	Yes	Yes	Yes	Yes
Adjusted *R*^2^	0.9769	0.9768	0.9769	0.9768	0.9787	0.8306
*N*	1669	1921	1807	1689	2598	1833

*** represent the significant levels of 1%. Parentheses indicate standard errors clustered at the county level. The baseline control variables are consistent with column (4) in Table 2.

#### Adopting county-level data

As mentioned in the above sections, the digital finance index covers data at the provincial, municipal, and county levels, so we adopt county-level data for robustness tests. However, the county-level data are not comprehensive in terms of control variables; therefore, we only include *GDP per capita, Fiscal expenditures, Fiscal revenue, Savings, Financial development, Number of college students*, and *Number of enterprises above the scale*, whose raw data are obtained from the China County Statistical Yearbook. All the DID estimates in Table 13 are significantly negative, except for column (2). In particular, the results in column (1) show that after 2013, for every 1 unit increase in the county-level digital financial index, the PM_2.5_ concentrations decrease by an average of 2.69%. Taken together, adopting county-level data, the conclusion that digital financial development significantly curbs haze pollution is not challenged.

**Table 13:** Robustness checks by adopting county-level data.

	**(1)**	**(2)**	**(3)**	**(4)**
DFI*Post13	−0.0269***			
	(0.0075)			
CBI*Post13		−0.0053		
		(0.0047)		
UDI*Post13			−0.0283***	
			(0.0074)	
DLI*Post13				−0.0157***
				(0.0036)
Baseline controls	Yes	Yes	Yes	Yes
County FE	Yes	Yes	Yes	Yes
Year FE	Yes	Yes	Yes	Yes
Province-by-year FE	Yes	Yes	Yes	Yes
Adjusted *R*^2^	0.9777	0.9777	0.9778	0.9778
*N*	8723	8723	8723	8723

*** represent the significant levels of 1%. Parentheses indicate standard errors clustered at the county level. The baseline control variables are consistent with column (4) in Table 2.

## Conclusion and discussion

The environmental benefits of digital finance have attracted scholarly interest in recent years [e.g., Wan et al. (19); Wen et al. (29); Wang et al. (30); Yang et al. (31)], but the causal effect and potential mechanisms have been overlooked. In this paper, the DID model is constructed to identify the cause-and-effect relationship by using the exogenous shock from China's 1st year of digital finance in 2013. Moreover, this causal relationship between digital finance and haze pollution is further confirmed by placebo tests and IV estimations. Additionally, we test the mechanisms by which digital finance curbs haze pollution in China, including technological innovation, industrial upgrading, and green development.

The main findings are summarized below. First, we find that digital financial development significantly reduces haze pollution in Chinese cities, as evidenced by a set of robustness checks. According to the DID estimates, a one standard deviation increase in digital finance after 2013 would result in 0.2708 standard deviations reduction in PM_2.5_ concentrations. Second, digital finance can drive technological innovation, promote industrial upgrading, and contribute to green development, which may further reduce haze pollution. Third, heterogeneity analysis shows that compared to the coverage breadth of digital finance, the usage depth of digital finance has a greater marginal impact on haze pollution reduction. Moreover, cities north of the Qinling-Huaihe Line, those with better Internet infrastructure, and those with higher levels of traditional financial development all benefit more from digital finance. The regression results show that for cities with light and very heavy haze pollution, the positive effect of digital finance is limited.

Our findings have important policy implications. First, our study provides evidence that digital finance can reduce haze pollution. As a result, it is critical to encourage the development of digital finance and further exploit its environmental benefits. Governments could further extend these experiences to more countries and regions to exploit their environmental-friendly benefits by improving policies connected to the digital finance and technology finance. Second, using digital finance to cut down on haze pollution depends on technological innovation, industrial upgrading, and green development. Thus, it is necessary to strengthen investment in technological innovation, promote high-tech industries, and achieve sustainable development. Possible measures include actively introducing new green industries, extending the industrial innovation chain, and strengthening pollution control. Third, to reduce haze pollution, it is vital for some cities with weak Internet infrastructure and financial foundations to speed up the building of Internet infrastructure and boost local financial development by introducing digital finance.

There are some limitations to our study that have implications for future research. First, although we explore some mechanisms by which digital finance affects haze pollution, other mechanisms are still preferable to be further added, such as green consumption and production. Second, because the research perspective of this paper is macro-level, micro-evidence is lacking. It would be interesting to look into the impact of digital finance on micro-individual green behavior at the firm and resident levels. At last, the paper's DFI is based on data from Ant Financial and Alipay. Therefore, these indexes lack evidence from other digital finance tools and digital finance platforms, such as WeChat Pay and Jingdong Finance. In the future, with a better digital finance index, some inspiring conclusions might be drawn.

## Data availability statement

Publicly available datasets were analyzed in this study. This data can be found at: https://idf.pku.edu.cn/zsbz/index.htm; https://sites.wustl.edu/acag/datasets/surface-pm2-5/; https://www.yearbookchina.com/navibooklist-N2014050073-1.html.

## Author contributions

CZ: conceptualization, methodology, validation, and writing-original draft. BY: writing-review and editing, visualization, and funding acquisition. Both authors contributed to the article and approved the submitted version.

## Funding

This work was supported by the Fundamental Research Funds for the Central Universities (No. QCDC-2020-21, CXJJ-2021-444).

## Conflict of interest

The authors declare that the research was conducted in the absence of any commercial or financial relationships that could be construed as a potential conflict of interest.

## Publisher's note

All claims expressed in this article are solely those of the authors and do not necessarily represent those of their affiliated organizations, or those of the publisher, the editors and the reviewers. Any product that may be evaluated in this article, or claim that may be made by its manufacturer, is not guaranteed or endorsed by the publisher.

## Footnotes

^1^ https://www.aljazeera.com/news/2017/12/23/chinas-killer-smog-rolls-in

^2^Ant Financial Services Group is the parent company of Alipay, both of which are part of the Alibaba Group.

^3^The original data for DFI came from Alipay.

^4^ http://finance.sina.com.cn/money/bank/gsdt/2018-05-04/doc-ifzyqqiq9855109.shtml

^5^Source: https://sites.wustl.edu/acag/datasets/surface-pm2-5/.

^6^These estimated coefficients are also insignificant at the 10% level.

^7^The former is compiled by the Industrial Development Research Center, Fudan University, and the latter is led by Enterprise Big Data Research Center, Peking University.

^8^These factors include *GDP per capita, Fiscal expenditures, Education expenditures, FDI, Internet penetration rate*, and *Financial development*.
