# Supplementary material for: Haze pollution reduction in Chinese cities: Has digital financial development played a role?
Source: Front Public Health. 2022 Aug 24;10:942243. doi: 10.3389/fpubh.2022.942243 (PMC9449125; doi:10.3389/fpubh.2022.942243)
Supplement: Supplementary file 1 [file Table_1.docx]

**Appendix**

**Table A1. Definitions and descriptive statistics for mechanism variables**

| Variables | Definition | N | Mean | SD |
| --- | --- | --- | --- | --- |
| **Panel A. Technological Innovation** | |  |  |  |
| Patents granted | Total patents granted in the city (logarithmic) | 1904 | 4.9359 | 0.6403 |
| Green patents applications | Total green patents applications in the city (logarithmic) | 1508 | 2.0074 | 1.3689 |
| Green patents granted | Total green patents granted in the city (logarithmic) | 1506 | 1.1099 | 1.0860 |
| Green utility model patents applications | Total green utility model patents applications in the city (logarithmic) | 1508 | 2.1721 | 1.2700 |
| Green utility model patent granted | Total green utility model patents granted in the city (logarithmic) | 1506 | 2.1069 | 1.2531 |
| City Innovation Power Index | An aggregate index from the Industrial Development Research Center of Fudan University (divided by 100) | 1509 | 0.1196 | 0.3934 |
| City Innovation and Entrepreneurship Index | An aggregate Index from the Enterprise Big Data Research Center, Peking University (divided by 100) | 1904 | 0.5207 | 0.2808 |
| **Panel B. Industrial upgrading** | |  |  |  |
| Industrial structure index | Share of employed population in primary industry*1+share of employed population in secondary industry*2+share of employed population in tertiary industry*3 | 1888 | 2.4950 | 0.1484 |
| Ratio of tertiary industry to secondary industry | Tertiary sector employment divided by secondary sector employment in the city | 1920 | 1.3457 | 0.8350 |
| Tertiary industry efficiency | Total output value of tertiary industry divided by employed population in the city | 1714 | 0.1685 | 0.0811 |
| Manufacturing | Manufacturing employment divided by secondary industry employment in the city | 1920 | 0.5284 | 0.1888 |
| **Panel C. Green development** | |  |  |  |
| GTFP | Green total factor production calculated by the Malmquist-Luenbergen Index at the city level | 1250 | 1.0065 | 0.1511 |
| EC | Efficiency change calculated by the Malmquist-Luenbergen Index at the city level | 1250 | 1.0046 | 0.1323 |
| TC | Technology change calculated by the Malmquist-Luenbergen Index at the city level | 1250 | 1.0093 | 0.1447 |
