# Supplementary material for: Haze pollution reduction in Chinese cities: Has digital financial development played a role?
Source: Front Public Health. 2022 Aug 24;10:942243. doi: 10.3389/fpubh.2022.942243 (PMC9449125; doi:10.3389/fpubh.2022.942243)
Supplement: Supplementary file 2 [file Table_2.docx]

**Appendix**

**Table A2. Input and output indicators for GTFP**

| Variables | Definition |
| --- | --- |
| **Panel A. Input indicators** | |
| Physical capital | Physical capital stock in cities using the perpetual inventory method |
| Labor | Total employed population in cities |
| Land | The total area of built-up area in cities |
| Energy | Total electricity consumption in cities |
| **Panel B. Expected output** | |
| GDP | Constant price GDP after deflating |
| **Panel C. Non-expected output** | |
| Wastewater | Total industrial wastewater emissions in cities |
| SO_2_ | Total industrial sulfur dioxide emissions in cities |
| Soot | Total industrial soot emissions in cities |
